# Architecture and plasticity: optimizing plant performance in dynamic environments

**DOI:** 10.1093/plphys/kiab402

**Published:** 2021-10-29

**Authors:** Ronald Pierik, Christian Fankhauser, Lucia C Strader, Neelima Sinha

**Affiliations:** 1 Plant Ecophysiology, Department of Biology, Utrecht University, 3584 CH Utrecht, the Netherlands; 2 Center for Integrative Genomics, Faculty of Biology and Medicine, University of Lausanne, CH-1015 Lausanne, Switzerland; 3 Department of Biology, Duke University, Durham, North Carolina 27278, USA; 4 Department of Plant Biology, College of Biological Sciences, University of California, Davis, California 95616, USA

## Abstract

Plasticity in plant architecture drives plant performance through dedicated molecular networks.

Most plants grow anchored to their substrate, but have an incredible ability to sense their environment and develop accordingly. Plant architecture refers to the 3D organization of the plant phenotype and is a key determinant of resource acquisition. Plasticity of this architecture allows plants to modify their architecture when the environment changes and calls for a reorganization of their development. How would architecture determine resource acquisition? Picture a natural grassland. Many plant species coexist but also compete for resources. Some species may be fast to emerge and produce a display of leaves to capture sunlight. Others that arrive slightly later need to elongate their stems to escape from the shade cast by the first layer of leaves. These would typically display increased apical dominance, that is, suppress shoot branching, to foster rapid stem elongation (Casal, 2013). Below ground, the soil is not homogeneous, and roots will be positioned where nutrient supply is optimal. Plants do this through regulation of root branching such that dense packages of root tissue can be formed in nutrient-rich zones, whereas branching is suppressed in nutrient-poor conditions (Drew, 1975). Plant architecture ensures that organs for resource acquisition are positioned where resource availability is optimal. Since the environment is highly dynamic, architecture must be plastic as well, hence, the overwhelming developmental flexibility displayed by plants. In this Focus Issue, six *Updates* review our current understanding of both above- and below-ground aspects of plant architecture and its plasticity. Research articles published with this issue and those published that appeared a bit earlier provide novel insights into a variety of signaling and response pathways that modulate plant architecture.

Shoot and root branching, as well as tuberization in some species, are regulated by genetically encoded developmental programs, yet are highly flexible in response to the environment (Karlova et al., 2021; Kondhare et al., 2021; Luo et al., 2021; Pélissier et al., 2021). These processes rely strongly on meristem transitions (Gaarslev et al., 2021) to form inflorescences, axillary buds and lateral root primordia. These genotype-environment interaction-driven branching and phase transition processes help plants create an optimal architecture for resource acquisition. However, it is not only branching and meristem fate that determine architecture; existing organs can also optimize their 3D position and biochemistry in response to their local environment (Furutania and Morita, 2021; Shi and Liu, 2021). Shoot branching is perhaps the most noticeable aspect of plant architecture as it is not hidden in the ground and determines the overall visible appearance of a plant. Branches arise from axillary buds, which in turn are initiated from activated axillary meristems. Luo et al. (2021) review the molecular and genetic networks that control axillary meristem activation and axillary bud outgrowth. In this review, the authors propose a combinatory network of hormones, transcription factors, and miRNAs that integrate environmental input with developmental branching programs. They argue that although model species have greatly improved our mechanistic understanding of shoot branching, there is an urgent need to both diversify our knowledge and translate it to crop species. Kondhare review how axillary meristems in potato do not only form shoot branches but can also change fate to develop tubers from modified belowground stems, called stolons. They describe the basic principles of tuberisation, including the molecular mechanisms involved, as well as as how this phenomenon is controlled by photoperiod (Khondare et al., 2021).

The task of the vegetative shoot is primarily to power the plant with energy harvested in the process of photosynthesis and plants have evolved extensive signaling systems to explore light cues to optimize shoot architecture. Shi and Liu (2021) review how UV-B light is both a stress factor as well as a signal for the light environment leading to adaptive plastic responses. In response to UV-B stress, plants produce ‘sunscreens’, protectants such as flavonoids, against UV-B damage. The authors review how some of the responses involve the UV-B receptor UV-B resistance 8 (UVR8), whereas UVR8-independent pathways are also identified. In addition, interactions with photoreceptors for lights of other wavelengths are discussed. One such group of photoreceptors is the phytochromes that are differentially sensitive to red and far-red light. Phytochromes directly control the activity of a family of basic helix–loop–helix (bHLH) transcription factors known as phytochrome interacting factors (PIFs). PIF activity is inhibited by phytochrome B (phyB) in the sun and shade releases this inhibition leading to PIF-mediated elongation of stem-like structures (Casal, 2013). This growth mechanism is tightly controlled as PIFs also promote expression of inhibiting HLH factors, which prevents excess PIF activity.

Buti et al. (2020) identify another layer of this growth regulation by showing that the KIDARI bHLH factor inhibits some of those inhibitors through protein–protein interactions. This shows that an elaborate transcriptional “gas and brake” mechanism underlies shoot growth in response to shade (Buti et al., 2020). Although shade promotes the elongation of stems and petioles, it restricts expansion of leaf blades. Romanowski et al. (2021) show that this restriction occurs through two distinct cellular strategies. Early in the shade response, cell division is limited while later cell expansion is reduced. Extensive gene expression analysis shows how phytochromes may regulate cell division and expansion at multiple levels (Romanowski et al., 2021).

These shade avoidance responses are known to be accompanied by repressed resistance against attackers; the growth-defense trade-off. Courbier et al. (2021) describe the kinetics of transcriptome reprogramming that is associated with the phytochrome-dependent suppression of the plant immune system, rendering tomato (*Solanum lycopersicum*) plants more vulnerable to attack by the fungal pathogen *Botrytis cinerea*. In Arabidopsis, phytochrome-mediated shade perception also accelerates flowering through a mechanism involving PIF-regulated expression of the floral promoters Flowering Locus T (FT) and Twin Sister of FT (Galvao et al., 2019; Zhang et al., 2019). Sun et al. (2021) show that in roses, low light delays flowering through a mechanism involving PIFs. The authors present evidence indicating that in low light PIFs interact with the floral regulator CONSTANS preventing it from inducing FT expression. Increasing light intensity leads to degradation of the PIFs thereby releasing CONSTANS inhibition and promoting flowering (Sun et al., 2021). Interestingly, in Arabidopsis low-light conditions elicit a so-called shade tolerance phenotype of the leaves that is mostly independent of the photoreceptors but resembles the phenotype of juvenile leaves (Xu et al., 2021). The authors studied whether low light delays the juvenile to adult transition. They determined that two major regulators of juvenile to adult phase transition, miR156 and miR157, are also critically involved in creating the low-light leaf phenotype, and that the canonical shade avoidance pathways are not involved.

In most plants, leaves are flat which favors light capture and gas exchange. Legris et al. (2021) show that the phototropin photoreceptors perceive light direction to control leaf flattening. This occurs during the cell expansion phase of leaf growth and depends on the establishment of an auxin signaling gradient across the leaf. Moreover, the authors provide evidence suggesting that controlling leaf flattening depending on the light environment contributes to photosynthesis optimization (Legris et al., 2021) . For optimal photosynthesis, the exact positioning of leaves inside the canopy is crucial. Batist et al. (2021) developed clip-on digital sensors to monitor leaf 3D positioning. They show how these devices can monitor leaf movements in 3D in real-time, allowing the researcher to distinguish horizontal and vertical leaf movements as well as torsions of the organ. The device is calibrated against conventional imaging-based data and proven to be able to record stress-induced changes in daily leaf angle rhythms. Such shoot measuring methods are instrumental for basic research and also for breeding. Breeding for optimal plant architecture and in particular leaf angles has led to substantial increase in yield, for example, in maize. Mantilla-Perez et al. (2020) present an interesting approach to optimize Sorghum architecture with a so-called “smart canopy” structure with leaves on top being more vertical while lower leaves have a more horizontal angle to favor light interception over the entire plant. Using GWAS and QTL studies they identify a potential role for the brassinosteroid hormones in the control of such “ideal” crop architecture (Mantilla-Perez et al., 2020).

In their review on meristem transitions, Gaarslev et al. (2021) provide an overview of the flowering transition and inflorescence architecture. They emphasize that transition to flowering typically releases the lateral buds from apical dominance, thus allowing short branching. The authors review how, for example in Arabidopsis, the transition to flowering is indeterminate, meaning that that apical meristem can continue to form lateral floral meristems until being inhibited or exhausted. In tomato on the other hand, the apical meristem is determinate and ends with a flower, whereas vegetative development continues from an axillary meristem that is activated from the leaf axil. The authors review how genetic components in this regulatory network have been targets for breeding optimal architectures for tomato yield. They emphasize how further fine-tuning holds promise for further improvement of vegetative versus reproductive architecture in tomato. In their research article, Walker et al. (2021) study inflorescence architectures in Arabidopsis and *Brassica napus*. They address the question of how different inflorescence architectures that remain flexible, can be enabled while a commitment to flowering has to be made very early in plant development, when plastic adjustments cannot yet be foreseen. They propose a feedback system, termed “integrative dominance”, to control flowering.

Goetz et al. (2021) focus their study in Arabidopsis on the question of how inflorescence growth and architecture are coregulated by fruit load. Inhibition of inflorescence growth starts while fruits are still developing, whereas it completely stops thereafter. The authors show that dominance inhibition of inflorescence growth by fruit load in Arabidopsis is mediated by a combination of sugar and auxin signaling. Yanga et al. (2021) focus on inflorescence architecture in the grasses. Here, the inflorescence can initiate from the inflorescence meristem, or after several branching events in the axillary meristems, allowing for strong variation of inflorescence architectures within the grasses. The authors identify a loss of function allele for AP1/FUL (FRUITFUL-LIKE) MADS-box transcription factor in *Setaria viridis* (green foxtail). They show that SvFUL2 drives not just inflorescence architecture but also flowering time in Setaria, and elucidate some of the associated molecular–genetic networks. This MADS-box transcription factor acts at the intersection of inflorescence architecture and flowering time, thus constituting a potentially important entry for optimization of cereal crop reproductive architecture.

In some cases, harsh environmental conditions pose a severe stress on plants. For example, when temperatures exceed ambient fluctuations, plants can perceive this heat stress and adjust to subsequent waves of heat through memory effects. Bi et al. (2021) study heat stress memory for heat stress tolerance in turfgrass. They show that the *Festuca arundinaceae* heat shock protein 17.8 Class-II displays a thermomemory for several days, affecting ROS and photosynthetic electron transport acclimations to heat. High temperature also influences reproduction and recombination which is a major motor of genetic innovations. Ning et al. (2021) studied the molecular mechanisms underlying inhibition of meiotic recombination by heat stress in Arabidopsis and find that such treatments inhibit crossover formation by interfering with double-strand breaks and synapsis formation.

Heat and drought stress are closely linked. In their study on durum wheat (*Triticum turgidum* spp. *durum*), Bacher et al. (2021) describe drought tolerance upon introgression of loci from the naturally drought-tolerant wild emmer *Triticum turgidum* spp. *dicoccoides*. The authors focused their imaging-based phenotyping analyses on root–shoot ratios and architecture and identified genetic loci associated with regulation of shoot–root carbon allocation under water deprivation. This provides opportunities toward improved water stress adaptation in crops, both for carbon allocation as well as for shoot and root architecture. How drought affects different growth stages and grain filling (yield) in maize was investigated by (Verbraeken et al., 2021). The authors used state-of-the-art phenotyping approaches to define the impact of water deficit at different growth stages throughout development. They define different drought-sensitive biomass yield components which should help for targeted breeding approaches.

Roots can take many different shapes, depending on interactions between genetic programs and microenvironmental conditions. Shao et al. (2021) performed a genetic screen by imaging rice roots in a hydrogel. They identified a regulator controlling root architecture (shallow versus deeper root systems) in rice and Arabidopsis. Field trials in rice indicate that a shallow root system results in no yield penalty, suggesting that manipulating this trait may allow one to identify variants better suited for specific soil conditions (Shao et al., 2021). Pélissier et al. (2021) review the molecular–genetic programs underpinning lateral root formation. Since lateral root formation is very strongly regulated by nutrient availability, and determines a plant’s access to these nutrients, the authors focus their review on developmental plasticity of lateral root formation. They outline the general pattern of nutrient interactions with root development, and outline in great detail how nitrate signaling regulates lateral root positioning.

Root branching is not just responsive to resource availability in order to maximize resource capture, but is also highly sensitive to environmental stress. Karlova et al. (2021) review the current understanding of how a multitude of abiotic stresses affect root system architecture and how root architecture may determine resilience against many of these abiotic stresses. In doing so, the authors also highlight the basic physiological and molecular networks underpinning root architectural plasticity under abiotic stress. They emphasize that in addition to adjusting root anatomy, growth, and branching, plant roots are also able to direct their growth direction away from stressful conditions and towards more optimal soil micro conditions. Indeed, this is also a well-known general aspect of root development relative to gravity, allowing roots to track gravity. Furthermore, lateral roots emerge at specific angles relative to the gravity vector, the gravitropic setpoint angle. Furutania and Morita (2021) show root gravitropic setpoint angle is regulated by LAZY-LIKE proteins involved in gravity signaling. The direction of root growth relative to the gravity vector determines how deep or shallow roots explore the soil volume. The authors review how LAZY-LIKE proteins that are expressed in gravity-sensing cells, statocytes, modulate auxin transport between the upper and lower side of the lateral root tip to regulate root angles relative to the gravity vector.

Many insights on root developmental plasticity have been obtained from studies on agar-based vertical plate systems in which root systems can be easily imaged. However, real soil is arguably more heterogeneous, dynamic, and complex than a homogeneous agar-based growth medium. Aguilar et al. (2021) developed a sensor tool, called RootTracker, to dynamically monitor root architecture and its plasticity under field soil conditions. The system is based on a multitude of sensors that are buried in the soil and detect roots that have approached a sensor. The authors were able to not only derive root growth and distribution, but also responses to drought stress, of maize plants under field conditions.

In closing, plasticity of plant architecture is at the basis of plant performance in natural as well as agricultural environments. The collection of *Updates* in this issue provides an excellent synopsis of our current understanding of the functional significance of plant architecture as well as the regulatory pathways that modulate it. The *Research articles* in this issue advance our understanding from this point onwards, and two *Breakthrough technologies, tools, and resources* contributions present technologies to help further this field of research. Integrative studies using phenotyping, genetics, and molecular physiology will both deepen our knowledge of the underlying developmental and physiological mechanisms, and allow translation of knowledge towards crop improvement in rapidly changing environments.

*Conflict of interest statement*. None declared
